# Analysis of Photosynthetic Parameters, Yield, and Quality Correlations in Herbicide-Tolerant Transgenic Hybrid Cotton

**DOI:** 10.3390/ijms27010400

**Published:** 2025-12-30

**Authors:** Ping He, Meiqi Liu, Haoyu Jiang, Zexing Zhang, Zitang Bian, Yongqiang Liu, Honglei Ma, Jianbo Zhu, Tianqi Jiao, Ruina Liu

**Affiliations:** 1 College of Life Sciences, Shihezi University, Shihezi 832003, China; heping1@xjshzu.com (P.H.); zq03117510@163.com (M.L.); yu65007780@163.com (H.J.);; 2 Crop Research Institute, Xinjiang Academy of Agricultural Sciences, Urumqi 830091, China

**Keywords:** *Gossypium hirsutum*, *GAT* gene, *GR79-EPSPS* gene, glyphosate resistance, heterosis, gene expression regulation, photosynthetic performance, oxidative stress

## Abstract

Weed stress remains a major limiting factor in cotton production, and glyphosate-tolerant varieties provide an effective solution for chemical weed control. However, achieving a balance between herbicide tolerance and agronomic physiological traits remains challenging. In this study, three hybrid combinations were generated by crossing a glyphosate-tolerant cotton line (GGK2) with conventional elite lines and were comprehensively evaluated. Gene expression analysis revealed that the classical detoxification gene *GAT* was significantly downregulated in all hybrid combinations, whereas the expression of *GR79-EPSPS*, a gene associated with glutathione metabolism and oxidative stress response, was markedly elevated, particularly in the GGK2 × Y4 combination. This differential expression pattern suggests that *GR79-EPSPS* may compensate for the reduced function of *GAT* by conferring oxidative protection under herbicide stress. Physiological determination indicated that hybrid combinations with enhanced *GR79-EPSPS* expression, especially GGK2 × Y5, exhibited superior photosynthetic pigment composition and photosystem II (PSII) efficiency, validating the role of *GR79-EPSPS* in maintaining photosynthetic stability. Agronomic trait assessment demonstrated that GGK2 × Y4 achieved significant biomass accumulation and yield improvement through heterosis, although fiber quality improvement was limited. This study effectively enhanced the herbicide resistance of conventional cotton through crossbreeding and revealed that the interaction between *GR79-EPSPS* and GAT can improve cotton tolerance to herbicides, thereby providing a breeding strategy for developing cotton varieties with both herbicide tolerance and superior agronomic traits.

## 1. Introduction

Weed infestation in agricultural fields represents one of the primary factors contributing to crop yield reduction. Traditional manual weeding methods are not only labor-intensive and inefficient but also increasingly incompatible with the large-scale, mechanized operations characteristic of modern agriculture [1,2,3]. The extensive application of herbicides has significantly suppressed weed growth and improved crop management efficiency; however, it has also exerted certain adverse effects on crop growth and development [4]. Since 1996, genetically modified (GM) crops with glyphosate resistance have been successively developed, including glyphosate-resistant soybean and rapeseed (1996), cotton (1997), maize (1998), and sugar beet (1999) [5,6]. Herbicide-tolerant crops account for more than 94% of the 180 million hectares of GM crops cultivated globally each year, with glyphosate-resistant soybean, maize, cotton, rapeseed, and sugar beet collectively occupying approximately 80% of the total planted area [7]. The widespread adoption of herbicide-resistant GM crops has substantially reduced the frequency and overall volume of herbicide applications worldwide, while simultaneously lowering labor costs associated with manual weeding in agricultural production [3]. Nevertheless, this has also led to a significant decline in weed species diversity within agroecosystems, intensified reliance on single herbicides such as glyphosate, and increased the potential risk of herbicide-resistant weed evolution [8].

Upland cotton *(Gossypium hirsutum L.)* is one of the most economically important crops globally, valued for its fiber and oil. In recent years, however, global cotton cultivation area has exhibited a declining trend due to multiple factors, including rising production costs, natural resource constraints, and threats from pests and diseases [9,10,11,12]. According to FAO statistics, the cultivation areas in China, India, the United States, and Brazil—the four leading cotton-producing countries—were 3250.00, 1347.70, 3348.61, and 1633.42 thousand hectares in 2020, with corresponding yields of 5511.10, 1315.70, 2748.80, and 4329.30 thousand metric tons, respectively. By 2022, the cultivation areas in China, India, and the United States had decreased by 7.69%, 8.20%, and 10.08% year-on-year, respectively. Although slight yield increases were observed in some countries (e.g., China: +9.61%; United States: +2.31%), an overall fluctuating downward trend has been evident. In the context of the increasingly prominent global imbalance between cotton supply and demand, reliance solely on conventional breeding methods is insufficient to meet the demand for high-quality, high-yield raw materials required by the modern textile industry. Therefore, accelerating the development of new cotton varieties with high yield, superior quality, and stress resistance has become particularly urgent.

Among various breeding strategies, heterosis represents one of the effective approaches for enhancing crop yield and stress tolerance. Studies have shown that F_1_ hybrid varieties of crops generally achieve approximately a 20% increase in yield, while the F_2_ generation can still maintain an advantage exceeding 10% [13]. Furthermore, hybrid lines across multiple generations exhibit favorable genetic stability in terms of adaptability, biomass accumulation, and yield performance. However, the utilization of heterosis remains significantly constrained due to the strong randomness in cross-combination screening, prolonged breeding cycles, and high costs associated with hybrid breeding. The rapid advancement of molecular breeding technologies, particularly transgenic technology and gene editing, has opened new avenues for improving stress tolerance and enabling precision breeding in cotton [14,15,16,17]. Previous studies have confirmed that transgenic cotton varieties carrying insect-resistant, drought-tolerant, or herbicide-tolerant genes demonstrate satisfactory performance in expression efficiency, environmental adaptability, and yield stability [18,19,20,21]. Nevertheless, limitations such as current transformation efficiency, stability of target site expression, and screening of functional genotypes continue to pose substantial obstacles to cotton breeding progress in China [22,23,24].

Glyphosate is a highly effective and broad-spectrum herbicide; however, it kills all plants (including crops) because it inhibits the activity of a key enzyme (EPSPS) in plants and microorganisms, thereby preventing the synthesis of essential aromatic amino acids. The research team led by Guo Sandui employed metagenomic techniques to isolate two key glyphosate-resistance genes from bacteria in soil that had been heavily contaminated with glyphosate over a long period: the *GR79-EPSPS* gene, which encodes an EPSPS enzyme variant insensitive to glyphosate, and the *GAT* gene, which encodes glyphosate acetyltransferase, an enzyme that inactivates glyphosate through acetylation. Using Agrobacterium-mediated transformation, the constructed vector was introduced into cotton cells, and transgenic cotton seedlings were selected on a culture medium containing glyphosate. Transgenic plants were self-pollinated for multiple generations, and combined with continuous glyphosate spray screening, individuals with strong resistance and desirable agronomic traits were selected. Through methods such as PCR and Southern blot, it was confirmed that the target genes were stably integrated into the cotton genome and were expressed. Following the above rigorous procedures, the research team ultimately obtained a stable, glyphosate-resistant cotton line designated GGK2 [25,26]. However, the currently available transgenic glyphosate-resistant cotton materials still fail to meet production demands, and the resources of traditional elite cotton varieties have not yet been fully utilized. This study plans to use the GGK2 line as the paternal parent and cross it with conventional maternal varieties XH, Y4, and Y5, which possess superior yield and quality traits. The aim is to analyze the agronomic traits and glyphosate resistance of the hybrid progeny, thereby selecting new cotton resources that combine high yield, high quality, and herbicide resistance. This would enable farmers to safely apply glyphosate for weed control in cotton fields without harming the cotton plants themselves.

In summary, by utilizing glyphosate-tolerant transgenic cotton as the male parent and crossing it with conventional female parents possessing favorable yield and quality traits, it is possible to fully exploit heterosis for the development of high-yielding, high-quality, and glyphosate-resistant cotton varieties. A systematic comparison of these hybrid combinations in terms of herbicide resistance, field performance, photosynthetic capacity, yield components, and quality traits will provide a theoretical foundation and material resources for future commercial breeding of stress-tolerant transgenic cotton.

## 2. Results

### 2.1. Differential Expression of GAT and GR79 in Cotton Male Parents and Hybrids

**Figure 1:**
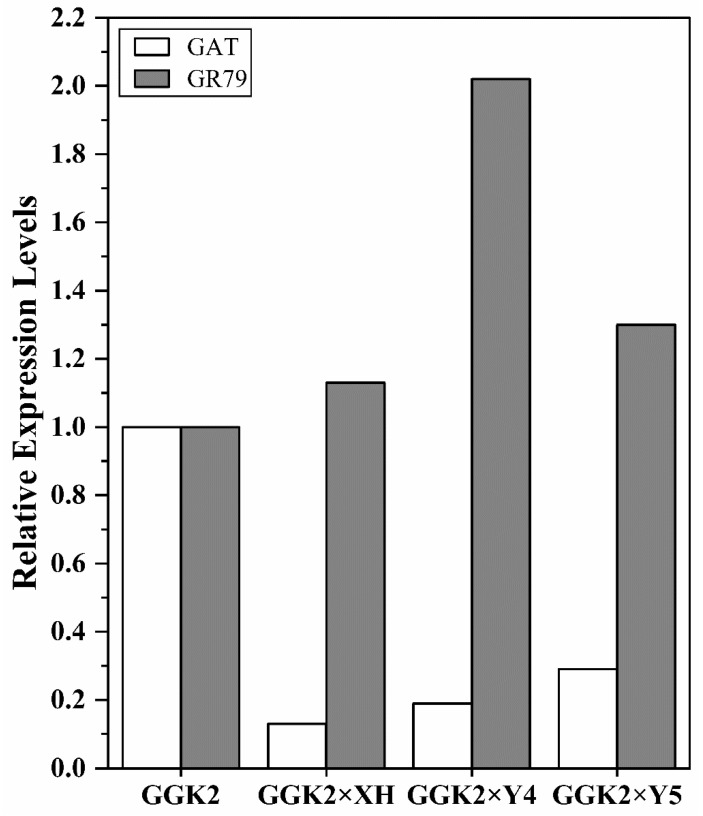
Elative expression levels of the *GAT* and *GR79* genes in the paternal and hybrid lines. (The relative expression level in the offspring was calculated by comparing the mean relative expression value of the offspring to that of the paternal group. Expression levels relative to those of the GGK2-XH plants were calculated by the 2^−ΔΔCt^ method (ΔΔCT = (CT, GGK2 × XH − CT, UBQ) − (CT, XH–CT, UBQ). Using the paternal mean value as the normalized standard (set to 1), the remaining two progeny were calculated using the same method.

To investigate the factors underlying the differences in herbicide tolerance among the hybrid lines, the relative expression levels of herbicide-related genes in the hybrids were analyzed. The primer sequences used are listed in Table 1. The data in Figure 1 revealed that, compared to GGK2, the expression of the *GAT* gene was downregulated in the hybrids, with reductions of 87%, 81%, and 71% in GGK2 × XH, GGK2 × Y4, and GGK2 × Y5, respectively. In contrast, the expression of GR79 was upregulated relative to GGK2, with increases of 13%, 102%, and 30%, respectively.

### 2.2. Phenotypic Responses of Parental and Hybrid Cotton Lines Under Herbicide Stress

**Figure 2:**
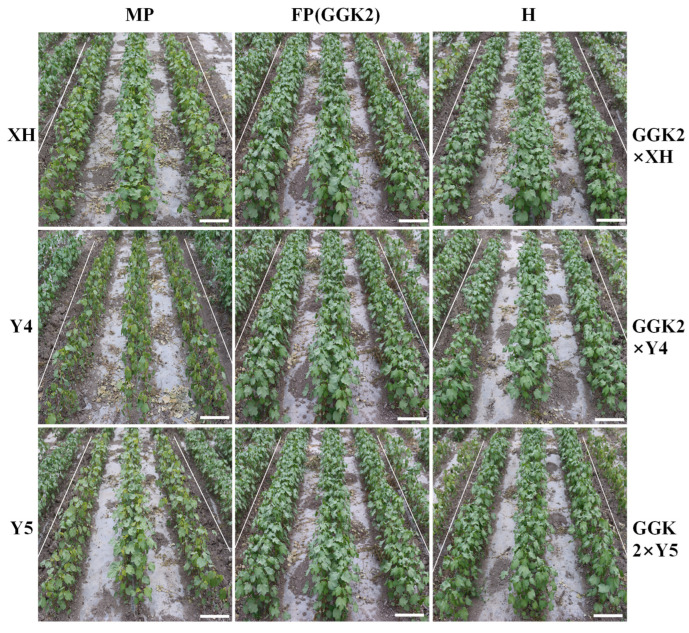
Field evaluation of three hybrid cotton combinations sprayed with 4500 g/ha glyphosate at the seedling stage. MP–XH, MP–Y4, MP–Y5, FP–GGK2, and H–XH, H–Y4, H–Y5, respectively. Scale bar, 20 cm.

As shown in Figure 2 under treatment with a 0.5% herbicide concentration, the paternal parent GGK2 and the three hybrid lines (GGK2 × XH, GGK2 × Y4, and GGK2 × Y5) exhibited herbicide tolerance. In contrast, herbicide injury symptoms were observed in the three maternal parents (XH, Y4, and Y5) following herbicide application, characterized by noticeable yellowing of the young leaves and light yellow discoloration on other true leaves.

### 2.3. Study on Photosynthetic Pigment Accumulation and PSII Performance in Herbicide-Resistant Cotton Hybrids During the Boll Period

**Figure 3:**
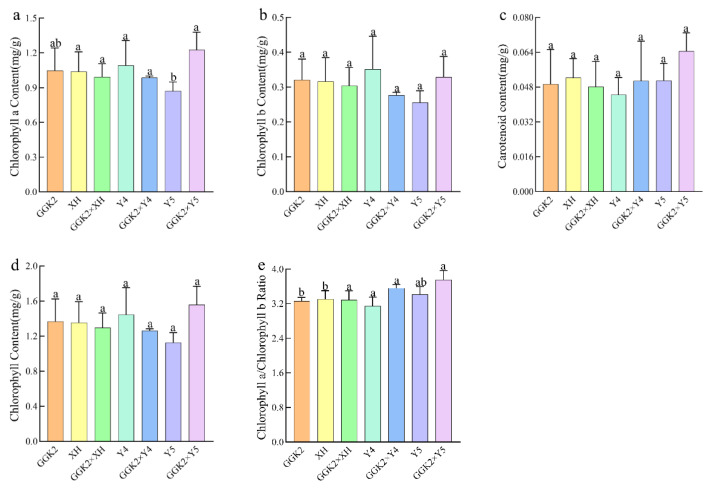
Determination of Pigment Content at the Boll Stage in Three Hybrid Combinations. (**a**). Determination of Chlorophyll a Content, (**b**). Determination of Chlorophyll b Content, (**c**). Determination of Carotenoid Content, (**d**). Determination of Total Chlorophyll Content, (**e**). Chlorophyll a/Chlorophyll b Ratio. Note: The abbreviations Chla, Chlb, ChlT, Chl a/b, and Car represent chlorophyll a content, chlorophyll b content, total chlorophyll content, chlorophyll a/b ratio, and carotenoid content, respectively. The letters a and b indicate significance at the 5% probability level. MPH denotes mid-parent heterosis, calculated as MPH = (trait value of hybrid − mean trait value of parents)/mean trait value of parents × 100%; SPH denotes super-parent heterosis, calculated as SPH = (trait value of hybrid − trait value of better parent)/trait value of better parent × 100%. A value greater than 0 indicates positive heterosis, while a value less than 0 indicates negative heterosis.

To determine whether herbicide tolerance genes introduced through hybridization affect pigment accumulation in cotton varieties, the contents of chlorophyll and carotenoids were measured in various germplasms during the boll-forming stage. As shown in Figure 3 and Table A1, a significant difference in chlorophyll a content was observed only in the germplasm GGK2 × Y5, while the pigment contents of the other germplasms showed no significant differences compared to their parental lines. This indicates that pigment content is minimally influenced by the *GAT* and *GR79* genes. However, in terms of heterosis, the hybrid germplasm GGK2 × Y5 exhibited relatively high MPH and SPH values for pigment traits. Specifically, MPH values of 28.08%, 14.09%, 24.84%, 12.51%, and 22.39% were observed for Chla, Chlb, ChlT, Chl a/b, and Car, respectively, while the corresponding SPH values were 17.34%, 2.49%, 13.86%, 9.86%, and 15.18%. Similarly, for the Chl a/b ratio, GGK2 × XH, GGK2 × Y4, and GGK2 × Y5 showed MPH and SPH values of 11.32% and 9.36%, 0.15% and –0.61%, and 12.51% and 9.86%, respectively.

There exists a close and multifaceted relationship between chlorophyll fluorescence kinetic parameters and the performance of photosystem II. Chlorophyll fluorescence kinetic parameters, which reflect the absorption, transfer, dissipation, and allocation of light energy by the photosystems during plant photosynthesis, can be utilized to quantify the mechanisms of plant photosynthesis and the physiological status of plants, thereby facilitating the screening of plant varieties with high photosynthetic efficiency.

**Figure 4:**
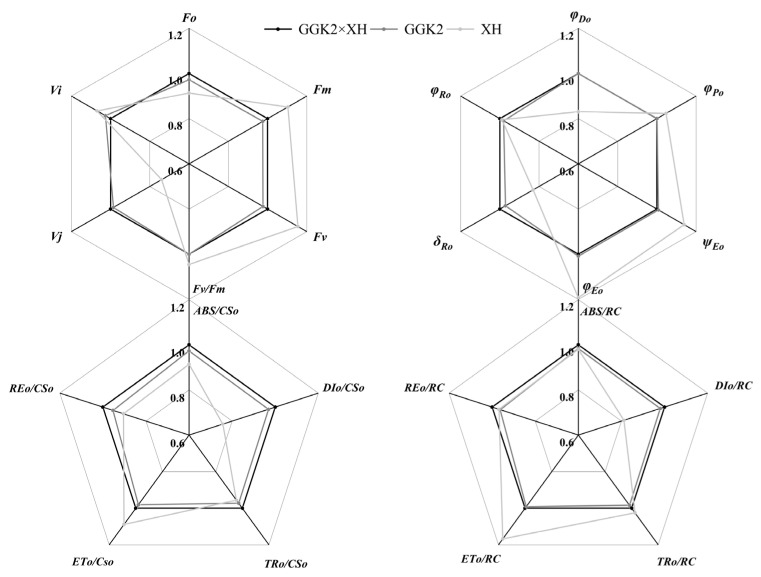
Comparative Analysis of Relative Differences in Chlorophyll Fluorescence Kinetic Parameters of Photosystem II Between GGK2 × XH and Its Parental Lines. (Fo: Minimal fluorescence (all PSII reaction centers open); Fm: Maximal fluorescence (all PSII reaction centers closed); Fv: Variable fluorescence = Fm − Fo; Fv/Fm: Maximum quantum yield of PSII photochemistry; Vj: Relative variable fluorescence at the J-step; Vi: Relative variable fluorescence at the I-step; φPo: Phi Po (Fv/Fm), maximum quantum yield of primary photochemistry in PSII; ψEo: Psi Eo (ET_0_/TR_0_), probability that an absorbed photon will move an electron into the electron transport chain beyond QA^−^; φEo: Phi Eo (ET_0_/ABS), quantum yield for electron transport from QA^−^ to the PSI end acceptors; δRo: Delta Ro (RE_0_/ET_0_), probability that an electron from the intersystem electron carriers is transferred to reduce PSI end acceptors; φRo: Phi Ro (RE_0_/ABS), quantum yield for reduction in PSI end acceptors per absorbed photon; φDo: Phi Do (DI_0_/ABS), quantum yield of energy dissipation per absorbed photon; ABS/CS_0_: Absorption flux per leaf cross-section; DI_0_/CS_0_: Dissipated energy flux per cross-section; TR_0_/CS_0_: Trapped energy flux per cross-section; ET_0_/CS_0_: Electron transport flux per cross-section; RE_0_/CS_0_: Electron flux from QA^−^ to PSI acceptors per cross-section; ABS/RC: Absorbed energy flux per reaction center; TR_0_/RC: Trapped energy flux per reaction center; ET_0_/RC: Electron transport flux per reaction center; RE_0_/RC: Electron flux to terminal acceptors per reaction center; DI_0_/RC: Dissipated energy flux per reaction center. The same applies below).

Figure 4 presents the relative values of chlorophyll fluorescence kinetic parameters for GGK2 × XH and its parents. It was observed that the chlorophyll fluorescence kinetic parameters of GGK2 × XH were relatively similar to those of the paternal parent GGK2, with nearly all parameter values being higher in GGK2 × XH than in GGK2. However, significant differences were noted compared to the maternal parent XH. For instance, in terms of quantum efficiencies or flux ratios, φPo, ψEo, and φEo were significantly higher in XH than in GGK2 × XH. Additionally, the initial light absorption per cross-section (ETo/CSo) and per reaction center (ETo/RC) were markedly higher in XH compared to GGK2 × XH. Conversely, other parameters of GGK2 × XH—namely φDo, δRo, ABS/CSo, DIo/CSo, TRo/CSo, REo/CSo, ABS/RC, TRo/RC, REo/RC, and DIo/RC—were all higher than those of the maternal parent XH.

**Figure 5:**
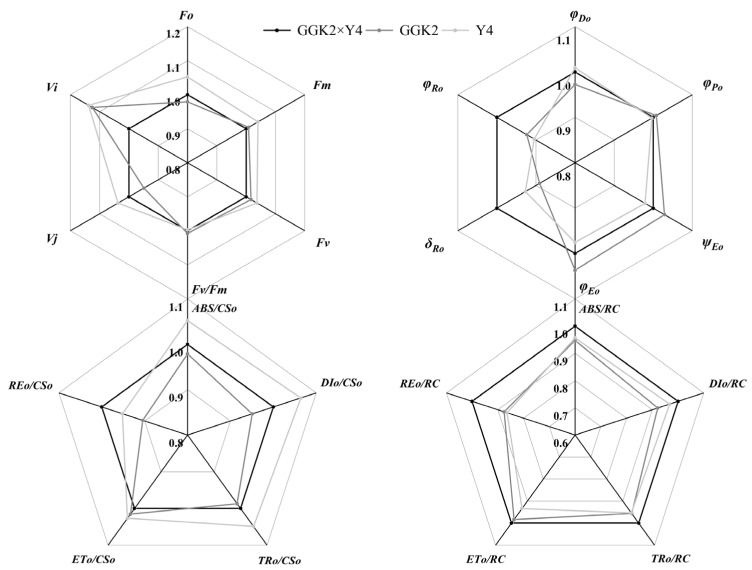
Comparative Analysis of Relative Differences in Chlorophyll Fluorescence Kinetic Parameters of Photosystem II Between GGK2 × Y4 and Its Parental Lines.

As illustrated in Figure 5, the differences between GGK2 × Y4 and its parents are primarily manifested in the quantum efficiencies or flux ratios and the initial apparent quantum flux per unit light-exposed cross-sectional area. Specifically, φRo, δRo, and REo/CSo of GGK2 × Y4 were significantly higher than those of its parents. In contrast, the maternal parent Y4 exhibited higher values for ABS/CSo, DIo/CSo, and TRo/CSo compared to GGK2 × Y4. Notably, all Structural Indicators & Fluxes parameters—namely ABS/RC, TRo/RC, ETo/RC, REo/RC, and DIo/RC—were higher in GGK2 × Y4 than in its parents. Additionally, the paternal parent GGK2 demonstrated higher ψEo and φEo values than the hybrid line.

**Figure 6:**
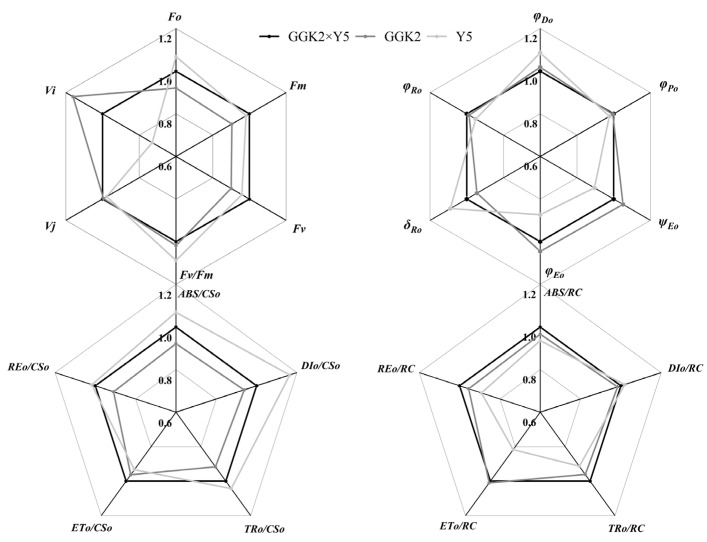
Comparative Analysis of Relative Differences in Chlorophyll Fluorescence Kinetic Parameters of Photosystem II Between GGK2 × Y5 and Its Parental Lines.

As shown in Figure 6, differences in chlorophyll fluorescence kinetics were primarily observed between the maternal parent and GGK2 × Y5. The values of φDo, δRo, ABS/CSo, DIo/CSo, and TRo/CSo in the maternal parent were significantly higher than those in GGK2 × Y5, whereas ψEo, φEo, ABS/RC, TRo/RC, ETo/RC, and REo/RC were lower than those in GGK2 × Y5. The chlorophyll fluorescence kinetic parameters of the paternal parent GGK2 were nearly identical to those of GGK2 × Y5.

### 2.4. Analysis of Biomass Distribution, Yield Performance, and Fiber Quality in Three Cotton Hybrid Combinations

To further understand the heterosis of the hybrid germplasms, the fresh weight and dry weight of each germplasm were measured. As shown in Table 2, among the three hybrid germplasms, only GGK2 × Y4 exhibited high heterosis for fresh weight across various tissues. It demonstrated MPH values of 51.07%, 41.07%, 43.18%, 125.16%, and 32.05%, and SPH values of 35.22%, 33.33%, 31.26%, 91.96%, and 18.36% for the whole plant, root, stem, leaf, and boll, respectively. GGK2 × Y5 exhibited MPH for fresh weight in all tissues (14.97%, 31.85%, 15.11%, 14.15%, and 15.42%, respectively), while SPH was observed only in the root and leaf (30.32% and 30.63%, respectively). In addition, GGK2 × XH also exhibited certain MPH (22.53% and 18.50%) and SPH (21.09% and 1.52%) in the root and leaf, respectively.

As shown in Table 3, significant differences were observed between the hybrid GGK2 × XH and its parents for root and leaf dry weights. This hybrid also exhibited relatively high MPH (42.87%, 35.83%) and SPH (36.42%, 21.09%) values for these traits. Significant differences were detected between the hybrid GGK2 × Y4 and its parents for plant, stem, and leaf dry weights. The MPH for GGK2 × Y4 ranged from 49.12% to 94.51%, while its SPH ranged from 31.17% to 84.04%. Similarly, the hybrid GGK2 × Y5 also demonstrated heterosis, with MPH ranging from 15.76% to 31.72% and SPH ranging from 9.33% to 31.22%.

As shown in Table 4, significant differences in agronomic traits at the boll stage were observed between the hybrid germplasms and their parents. In the cross GGK2 × XH, plant height and height to the first fruiting branch exhibited low MPH (2.46% and 6.06%, respectively), and their SPH values (−2.54% and −5.66%, respectively) indicated no heterosis. The number of bolls and number of fruiting branches both demonstrated certain levels of MPH (14.18% and 14.02%, respectively) and SPH (12.88% and 9.58%, respectively). No heterosis was observed for stem diameter. In contrast, the cross GGK2 × Y5 exhibited heterosis across all measured agronomic traits. Notably, high heterosis was observed for the number of bolls, number of fruiting branches, and stem diameter, with MPH values of 13.64%, 11.32%, and 9.43%, and SPH values of 13.64%, 5.99%, and 6.81%, respectively.

Yield traits are important indicators for crop evaluation. Table 5 displays the agronomic traits of the hybrid germplasms and their parents, as well as the heterosis rates of the hybrid germplasms. The cross GGK2 × XH exhibited heterosis in boll number per plant, yield per plant, and lint yield, particularly in boll number per plant, where its MPH and SPH were 2.98% and 21.47%, respectively. The heterosis observed in GGK2 × Y4 was similar to that of GGK2 × XH, with MPH values of 15.94%, 6.98%, and 6.98% for boll number per plant, yield per plant, and lint yield, respectively, and corresponding SPH values of 9.89%, 3.62%, and 10.56%. In contrast, GGK2 × Y5 demonstrated almost no heterosis.

The quality of cotton fiber determines the efficiency of textile processing. As shown in Table 6, significant differences in fiber quality were observed between the hybrid germplasms and their parents. The cross GGK2 × XH exhibited a certain level of heterosis for fiber elongation, specifically with MPH and SPH values of 18.51% and 15.37%, respectively. However, no heterosis was observed for the other quality parameters. Similarly, in GGK2 × Y4, only fiber elongation and micronaire value demonstrated low heterosis, with MPH values of 4.42% and 8.33%, and SPH values of 4.02% and 7.39%, respectively; no heterosis was detected for the remaining parameters. Furthermore, GGK2 × Y5 exhibited even lower heterosis, showing MPH values of 3.69%, 0.37%, 1.39%, and 7.35% for fiber length, uniformity, elongation, and micronaire value, respectively, but demonstrating SPH only for fiber length and micronaire value, at 1.60% and 6.64%, respectively.

## 3. Discussion

Glyphosate is a broad-spectrum herbicide widely used in agricultural production; however, its potential phytotoxicity limits the widespread adoption of elite crop varieties. The *GAT* gene, which can reduce glyphosate toxicity by catalyzing its N-acetylation, is one of the most commonly utilized herbicide-tolerant genes. In contrast, *GR79* is a relatively less-studied novel gene associated with herbicide tolerance, and its underlying mechanism is closely related to glutathione metabolism and oxidative stress response. Hybrid breeding is one of the most widely used and classical breeding methods in agricultural production. It involves crossing two or more parental lines with different desirable traits, followed by the selection of offspring that combine the advantages of both parents, thereby developing superior new varieties. Currently, hybrid breeding is often integrated with transgenic technology to enhance breeding efficiency [27,28,29].

The results showed that, compared to the paternal parent GGK2, the expression levels of *GAT* were significantly downregulated in three hybrid combinations (GGK2 × XH, GGK2 × Y4, and GGK2 × Y5), decreasing by 87%, 81%, and 71%, respectively. This may impair the *GAT*-mediated herbicide detoxification capacity. Notably, however, *GR79* was generally upregulated in the hybrid combinations, with increases of 13%, 102%, and 30%, respectively. In particular, its expression in GGK2 × Y4 was approximately twice that of the paternal parent. Based on previous studies indicating the critical role of GR-type genes in glutathione metabolism, ROS scavenging, and cellular protection [30,31,32], it is hypothesized that *GR79* plays an important function in alleviating glyphosate-induced oxidative damage. This regulatory role may be particularly prominent when *GAT* expression is suppressed. Furthermore, studies have suggested that co-expression of *GAT* and GR-type genes can enhance herbicide tolerance [25], which is consistent with the results of the field evaluation analysis in this study.

Analysis of herbicide-resistant phenotype was further consistent with the aforementioned results [25]. Under treatment with 0.5% glyphosate, all hybrid combinations exhibited a certain degree of resistance, indicating that the herbicide tolerance trait carried by the paternal parent GGK2 was successfully inherited in the hybrid germplasm and displayed a strong dominant or partially dominant inheritance trend [33,34,35,36,37]. The downregulation of *GAT* expression may be associated with the reorganization of cis- or trans-regulatory factors resulting from hybridization, whereas the high expression of *GR79* is likely driven by dominant alleles or enhanced effects of gene interactions, reflecting the integration of complex regulatory networks on target traits during hybridization [38]. Therefore, this study untangles, at the transcriptional level, that herbicide tolerance in hybrid varieties does not rely solely on the *GAT* gene, and suggests that novel resistance genes such as *GR79* may play a critical role in conferring resistance, thereby providing new targets and a theoretical basis for subsequent molecular breeding of herbicide-tolerant crops.

Abiotic stress conditions (e.g., salinity, drought, and heavy metals) generally exert significant impacts on the synthesis and distribution of photosynthetic pigments in plants [18,19,20]. In this study, based on the introduction of herbicide resistance-related genes *GAT* and *GR79*, we systematically analyzed changes in leaf pigment content and photosystem function across three hybrid combinations (GGK2 × XH, GGK2 × Y4, GGK2 × Y5) to explore the relationship between molecular regulation of herbicide tolerance and photosynthetic physiological performance. Regarding chlorophyll content, except for the significantly higher chlorophyll a (Chla) content in GGK2 × Y5 compared to its parents, no significant differences were observed between the other hybrid germplasms and their parents, indicating that altered expression of *GAT* and *GR79* did not inhibit pigment biosynthesis. However, further heterosis analysis revealed that GGK2 × Y5 exhibited significant mid-parent heterosis (MPH) and super-parent heterosis (SPH) in pigment accumulation, with MPH values for Chla, Chlb, and carotenoids (Car) reaching 28.08%, 14.09%, and 22.39%, respectively. Moreover, the Chla/b ratio in this combination was also significantly increased, suggesting a potential optimization of light capture and distribution efficiency through an elevated coordination ratio between chlorophyll a and b [39,40,41,42]. GGK2 × XH and GGK2 × Y4 also displayed a certain degree of heterosis in the Chla/b ratio. Such differences may be related to the maternal genetic background and its interactions with photosynthetic regulatory pathways, thereby influencing pigment synthesis efficiency and photosynthetic capacity.

To further validate the functional changes in photosynthesis regulation of the hybrid combinations, this study analyzed chlorophyll fluorescence kinetic characteristics, clearly untangling the response differences in the energy conversion pathway of photosystem II (PSII) among different genotypes. The fluorescence parameters of GGK2 × XH were overall similar to those of the paternal parent GGK2, and were higher than those of the maternal parent XH in most structural indicators (e.g., ABS/RC, TRo/RC, REo/RC), reflecting its strong foundation for light energy capture and electron transport. However, its photochemical efficiency indicators (e.g., φPo, ψEo, φEo) were lower than those of the maternal parent, indicating that despite superior structural performance, losses still occur in the light energy conversion process, potentially limited by gene interaction regulation [43,44,45]. GGK2 × Y4 exhibited outstanding performance in electron transport efficiency, particularly in indicators reflecting the depth of electron transport and the overall output efficiency of reaction centers, such as φRo, δRo, and REo/CSo, where it was significantly superior to both parents. This suggests that this combination can effectively deliver electrons to the terminal end of the electron donor chain, enhancing the energy output efficiency of the PSII system per unit area [44,46,47], further demonstrating its clear heterosis performance. Concurrently, its advantages in structural parameters of the reaction centers (e.g., ABS/RC, ETo/RC, REo/RC) indicate good potential for the structural integrity of the photosystem and the stability of electron output. In GGK2 × Y5, the fluorescence parameters were similar to those of the paternal parent GGK2, with some indicators such as ψEo and φEo being significantly higher than those of the maternal parent Y5, highlighting the genetic contribution of the paternal parent to its photosynthetic performance. Particularly noteworthy are the lower φDo and δRo values related to light energy dissipation in this combination, implying that higher electron transport efficiency is achieved based on lower energy loss, demonstrating a more economical and efficient light energy utilization characteristic, further supporting the overall advantages of this combination in pigment accumulation and photosynthetic efficiency [48,49,50]. Integrating the above results, it is evident that although the expression level of the herbicide-tolerance-related gene *GAT* was downregulated in some combinations, it did not cause significant inhibition of PSII system function. Conversely, in combinations with high *GR79* expression (e.g., GGK2 × Y5), the optimization of fluorescence parameters and the enhancement of photosynthetic structural stability were more pronounced, potentially related to the stress resistance-associated regulation involving *GR79* (e.g., ROS scavenging, cellular homeostasis maintenance). This physiologically reveals that *GR79* may play an auxiliary protective role in regulating the photosynthetic system, helping to maintain the photosynthetic activity of herbicide-tolerant hybrid combinations under adverse environmental conditions. These differences in photosynthetic function and pigment accumulation not only indicate varying response states of different hybrid combinations to herbicide-tolerance gene expression but also suggest a potential synergistic maintenance mechanism between herbicide-tolerance genes and photosynthesis regulation. This enhances the photosynthetic homeostasis capacity of hybrid combinations under abiotic stress conditions and also reflects the multi-level expression mechanism of heterosis in hybrid breeding. The potential synergistic maintenance mechanism between herbicide-tolerance genes and photosynthesis regulation further emphasizes the critical role of parental genetic background and its interaction with photosynthesis regulation in shaping the stress resistance and photosynthetic efficiency of hybrid cultivars.

Biomass accumulation and yield of hybrid germplasms are of widespread interest to researchers. In this experiment, significant combinatory differences were observed in the biomass accumulation of the hybrid germplasms, demonstrating strong dependence on genetic background. GGK2 × Y4 exhibited significant MPH and SPH in fresh weight indicators for the whole plant and various organs (root, stem, leaf, boll). The MPH and SPH for leaf fresh weight reached as high as 125.16% and 91.96%, respectively, indicating superior performance in photosynthetic product accumulation and organ-specific growth in this combination, potentially originating from higher leaf area index, photosynthetic efficiency, and dry matter conversion capacity. Although GGK2 × Y5 showed a certain degree of biomass advantage in roots and leaves, its overall advantage did not lead to systematic accumulation at the whole-plant level. In contrast, the biomass heterosis of GGK2 × XH was the most limited, being only slightly higher than the parents in some parameters of roots and leaves, suggesting relatively weaker capacity for carbon assimilate transport and inter-organ allocation [51]. Notably, regarding yield traits, GGK2 × XH exhibited low MPH or even negative SPH in individual structural traits such as plant height and height to the first fruiting branch, indicating no significant advantage in plant architecture construction. However, this combination still displayed moderate levels of heterosis in the number of fruiting branches and bolls, indicating enhanced lateral branch growth and reproductive establishment capacity [52,53]. The hybrid GGK2 × Y5 exhibited extensive heterosis in agronomic traits, particularly showing significant positive mid-parent heterosis (MPH) and specific combining ability (SCA) in multiple indicators such as the number of fruit branches, number of bolls, and stem diameter, indicating favorable field growth capacity. However, its heterosis for yield per plant was relatively low, which may be associated with reduced boll-setting rate or individual boll weight. These results suggest a certain degree of phenotypic dissociation between agronomic traits (e.g., plant height, stem diameter) and final yield-related traits, indicating that their correlation requires further optimization [54]. In terms of yield improvement, GGK2 × Y4 demonstrated the most prominent advantages, with MPH values reaching 15.94% for boll number per plant and 9.89% for yield per plant, making it the combination with the highest yield potential in this study. It is hypothesized that this hybrid has established a coordinated optimization pathway involving photosynthetic efficiency, biomass accumulation, and reproductive development rhythm, thereby providing a foundation for achieving high-yield objectives.

Although the aforementioned hybrid combinations exhibited varying degrees of superiority in biomass, agronomic traits, and yield, there remains considerable room for improvement in fiber quality. Overall, heterosis had limited effects on enhancing various fiber quality parameters (e.g., fiber length, strength, uniformity) [1,54,55]. Only GGK2 × XH showed a notable advantage in fiber elongation (MPH of 18.51%, SCA of 15.37%), while GGK2 × Y4 and GGK2 × Y5 exhibited minor improvements in parameters such as micronaire value. This indicates that, under the current parental combinations, hybridization breeding has not yet sufficiently improved the commercial properties of cotton. These findings suggest that fiber quality traits are governed by polygenic coordination, exhibit strong genetic stability, and low environmental sensitivity, making it difficult to achieve significant improvement through a single round of hybridization. The results imply that if fiber quality is the primary breeding objective, subsequent efforts should introduce high-quality fiber-type parents and incorporate marker-assisted selection to accelerate the pyramiding and optimized expression of superior fiber genes.

## 4. Materials and Methods

### 4.1. Plant Materials

Paternal parent: The GGK2 cotton variety was provided by the research team of Academician Guo Sandui (hereinafter referred to as GGK2). The optimal coding sequences (CDS) of the pGR79 EPSPS (1338 base pairs) and *pGAT* (441 base pairs) genes were commercially synthesized and co-cloned into the binary vector pBI121-CaMV 35S to generate the *pGAT-pGR79 EPSPS* construct, the restriction enzyme sites including Hind III, Bam HI, Xho I, and Eco RI, are indicated in the figure. Details regarding the gene sources and construction methods can be found in the literature by Guo et al. [25]. Maternal parents: Three local cotton cultivars widely cultivated in Xinjiang, namely Hexin 78, Xinluzao 33, and Xinluzao 78 (hereinafter referred to as XH, Y4, and Y5). All three cultivars were purchased from the agricultural supplies market in Shihezi City. Hybrid progeny: The F_4_ generation (GGK2 × XH, GGK2 × Y4, and GGK2 × Y5). The parental lines were conventionally cultivated in the experimental field of Shihezi University. The obtained F_1_ generation was planted in the experimental field and sprayed with glyphosate-isopropylamine salt at a concentration of 4500 g/ha to evaluate its herbicide resistance. Integration of the target genes into the progeny genome was confirmed by genomic PCR identification. Positive plants identified from the F_1_ generation were backcrossed to obtain F_2_ seeds. The F_3_ and F_4_ generation seeds were obtained using the same method as for the F_2_ generation. PCR and qRT-PCR analyses conducted on the F_4_ generation confirmed the presence of RNA expression for both the *GR79-EPSPS* and *GAT* genes in all three hybrid progeny populations.

### 4.2. Methods

#### 4.2.1. Quantitative Real-Time PCR

At the seedling stage of cotton, fresh leaves from the main stem (specifically the fourth leaf from the top after topping, i.e., the third leaf from the top post-topping) were collected, immediately placed in liquid nitrogen, and transported to the laboratory for storage in a −80 °C ultra-low temperature freezer. Total RNA was extracted from cotton leaves following the instructions of the FastPure Universal Plant Total RNA Isolation Kit (Vazyme, Nanjing, China). Using RNA as the template, first-strand cDNA was synthesized with the reverse transcription kit EasyScript^®^ One-Step gDNA Removal and cDNA Synthesis SuperMix (TRAN, CHAIN, Shanghai, China). Specific primers were designed using Primer-BLAST (https://www.ncbi.nlm.nih.gov/tools/primer-blast/, accessed on 21 December 2025) in the NCBI database based on the sequences of key herbicide-resistance-related regulatory genes in *Gossypium hirsutum*, such as *GAT* and GR79, as well as the cotton internal reference gene GhUBQ7. The expression levels of target genes were detected via quantitative real-time polymerase chain reaction (qRT-PCR) using a Bio-Rad real-time fluorescence quantitative instrument and SYBR Green Real-Time PCR Master Mix (KAPA Biosystems, Wilmington, DE, USA). The reaction volume was 10 μL, and the RT-PCR protocol was as follows: 95 °C for 5 min (1 cycle), followed by 40 cycles of 95 °C for 10 s, 59 °C for 15 s, and 72 °C for 10 s. Relative expression levels were calculated using the 2^−ΔΔCt^ method [56].

#### 4.2.2. Seedling Herbicide Tolerance Assay

This study was conducted during the 2023–2024 growing season at the transgenic safety assessment field of the Agronomy Experiment Station, Shihezi University, Xinjiang (45°19′ N, 86°03′ E). The experimental site is located in the middle section of the northern foothills of the Tianshan Mountains, characterized by a temperate continental climate. The mean annual temperatures for 2023 and 2024 were 9.10 °C and 8.97 °C, respectively, with annual precipitation ranging from 128.90 to 265.40 mm. The experiment was performed under controlled conditions using a drip irrigation system, arranged in a randomized complete block design with three replications. Each plot consisted of a 5 m row with an area of 10 m^2^, with row and plant spacings of 76 cm and 10 cm, respectively. Two seeds were sown per hill and thinned to one plant per hill at the seedling stage. All field management practices, including irrigation, fertilization, and pest and disease control, followed local standard agronomic protocols [57]. Based on a pre-determined seedling herbicide tolerance concentration [58], cotton plants at the seedling stage were treated with a commercial glyphosate-isopropylammonium formulation at a rate of 4500 g/ha. Concurrently, the growth phenotypes of cotton plants following herbicide application were photographed to document their growth status in response to the treatment for field evaluation. Based on the predetermined herbicide tolerance concentration for seedlings.

#### 4.2.3. Chlorophyll Pigment Content and OJIP Fluorescence

At the boll stage, the pigment content of the plants was determined. From each planting plot, healthy plants showing no signs of pest or disease damage were selected. Twenty leaf discs were collected from the fourth leaf from the top of the main stem (the third leaf from the top after topping) using a 1 cm diameter punch. These discs were immersed in sealed test tubes containing 10 mL of 95% ethanol for extraction. After the leaf discs turned white, the absorbance of the extracts was measured at 470 nm, 645 nm, and 663 nm using a spectrophotometer. The contents of chlorophyll a (Chl a), chlorophyll b (Chl b), total chlorophyll (Chl T), and carotenoids (Car) were calculated using the following formulae:

Chl a (mg/g) = 12.71 × OD663 − 2.59 × OD645(1)

Chl b (mg/g) = 22.88 × OD645 − 4.67 × OD663(2)

Chl T (mg/g) = Chl a + Chl b(3)

Car (mg/g) = 4.7 × OD470 − 0.27 × Chl T(4)

Also at the boll stage, OJIP fluorescence parameters were measured for plants in each plot. Five consecutive plants were selected per plot. The fourth leaf from the top of the main stem (the third leaf from the top after topping) was dark-adapted for 20 min between 10:00 AM and 12:00 PM. The fast chlorophyll a fluorescence induction kinetics curve (OJIP curve) was then recorded using a Handy PEA fluorometer. The measurements were conducted under an LED light source intensity of 3000 μmol·m^−2^·s^−1^, with a detection duration of 1 s for rapid fluorescence signal acquisition. To minimize the effects of leaf heterogeneity, three different points were measured on each leaf, and the average value was taken as the final rapid fluorescence data.

#### 4.2.4. Agronomic Traits, Yield Components, and Fiber Quality

At the boll stage, ten consecutive plants with uniform growth were selected for the analysis of agronomic traits. Plant height, height to the first fruiting node, number of fruiting branches, number of bolls, leaf area, internode length, and fruiting branch length were measured. Whole plants were carefully uprooted, After manually removing excess soil from the roots to avoid the influence of soil weight on the research results, the fresh weights of the whole plant, roots, stems, leaves, and bolls were measured separately. Each tissue was then deactivated at 105 °C and subsequently dried at 80 °C until a constant weight was achieved. The dry weight of each component was recorded, and the whole-plant dry weight was calculated by summing the dry weights of all components. Five replicates were performed for each variety.

Yield and yield components were assessed at maturity (October 15). From each plot, ten consecutive plants with fully open bolls were harvested. The seed cotton weight per plant was measured, and ginning was conducted under laboratory conditions to determine the lint percentage. Lint index was calculated based on lint percentage and seed index. Additionally, 50 fully open bolls from the middle portion of the plants were collected and weighed to determine boll weight. The total seed cotton weight from all fully open bolls in the plot was recorded as the final yield. Five replicates were assessed for each cultivar.

After ginning, a 30 g fiber sample from each plant was analyzed for fiber quality parameters, including fiber length, uniformity index, breaking tenacity, elongation at break, and micronaire value. These analyses were performed at the Institute of Agricultural Quality Standards and Testing Technology, Xinjiang Academy of Agricultural Sciences. Five replicates were assessed for each cultivar.

#### 4.2.5. Statistical Analysis

Data were tested for normality and homogeneity of variance using SPSS 26.0. A one-way analysis of variance (ANOVA) was conducted, and means were separated using the *T*-test at a significance level of 0.05. Figures were generated using Origin 2021 Pro. All data are presented as the mean ± standard error (SE) from at least three replicates.

## 5. Conclusions

Through integrated analysis of gene expression regulation, physiological parameters, and multi-level phenotypic data, this study untangled the differential responses of hybrid combinations across multiple dimensions—including pigment accumulation, biomass formation, agronomic traits, yield performance, and fiber quality—under the context of herbicide-tolerant gene introduction. Particularly in combinations with enhanced GR79 expression, not only was a strong physiological adaptability observed in pigment accumulation and photosystem stability, but promising growth and yield potential was also demonstrated. The spatial and organ-specific distribution of heterosis across these traits, along with its combination-dependent nature, provides theoretical support for further exploitation of elite hybrid germplasm. Moreover, it highlights potential trade-offs among different traits, underscoring the need for balanced and precise selection in subsequent breeding efforts to achieve coordinated optimization of yield, quality, and resistance.

Through integrated analysis of gene expression regulation, physiological parameters, and phenotypic data under glyphosate treatment, this study untangles the differential responses of hybrid combinations across multiple dimensions—including pigment accumulation, biomass formation, agronomic traits, yield performance, and fiber quality—in the context of herbicide-tolerant gene introduction. Particularly in combinations with enhanced GR79-EPSPS expression, not only was a strong physiological adaptation in pigment accumulation and photosystem stability observed, but promising growth and yield potential was also demonstrated. In this study, GR79-EPSPS and *GAT* were introduced into XH, Y4, and Y5 via hybridization, which enhanced the herbicide resistance of these three conventional cotton varieties while simultaneously exploiting heterosis, thereby enabling the development of high-yielding, high-quality, and glyphosate-tolerant cotton cultivars.

## Figures and Tables

**Table 1:** Primer involved in the experiment for RT-PCR.

Primer Name	Primer 5′→3′
RT GAT-F	AAGCAAGGAGGAGTGGTTGC
RT GAT-R	TCTTGCCTCCGATGAACTTG
RT GR79-F	TGATGGAGACCATGAGAGTG
RT GR79-R	CTCCAGGAAGTTGTCTGGTG
RT-GhUBQ-F	AGAGGTCGAGTCTTCGGACACC
RT-GhUBQ-R	TGCTTGATCTTCTTGGGCTTGG

**Table 2:** Fresh weight of various tissues at the boll stage.

Plant Lines	Fresh Weight (g)				
	Plant	Roots	Stems	Leaves	Bolls
GGK2	449.73 ± 41.25 a	14.07 ± 1.66 a	72.50 ± 8.85 a	83.30 ± 10.05 a	265.87 ± 34.55 a
XH	330.00 ± 34.48 b	13.74 ± 2.46 a	65.03 ± 6.11 a	59.43 ± 6.99 a	182.80 ± 19.70 ab
GGK2 × XH	350.80 ± 14.10 ab	17.03 ± 1.53 a	65.47 ± 4.84 a	84.57 ± 7.81 a	173.73 ± 2.32 b
MPH	−10.02%	22.53%	−4.80%	18.50%	−22.56%
SPH	−22.00%	21.09%	−9.70%	1.52%	−34.65%
GGK2	449.73 ± 41.25 ab	14.07 ± 1.66 a	72.50 ± 8.85 a	83.30 ± 10.05 b	265.87 ± 34.550 a
Y4	355.37 ± 33.93 b	15.80 ± 2.35 a	60.43 ± 7.80 a	58.73 ± 11.18 b	210.73 ± 16.71 a
GGK2 × Y4	608.13 ± 84.09 a	21.07 ± 3.61 a	95.17 ± 14.40 a	159.90 ± 22.26 a	314.67 ± 44.72 a
MPH	51.07%	41.07%	43.18%	125.16%	32.05%
SPH	35.22%	33.33%	31.26%	91.96%	18.36%
GGK2	449.73 ± 41.25 a	14.07 ± 1.66 a	72.50 ± 8.85 a	83.30 ± 10.05 a	265.87 ± 34.55 a
Y5	322.97 ± 17.590 b	14.40 ± 0.92 a	49.70 ± 2.86 a	64.63 ± 3.98 a	185.23 ± 9.48 a
GGK2 × Y5	444.20 ± 5.65 a	18.77 ± 1.23 a	70.33 ± 5.64 a	84.43 ± 5.66 a	260.33 ± 15.00 a
MPH	14.97%	31.85%	15.11%	14.15%	15.42%
SPH	−1.23%	30.32%	−2.99%	30.63%	−2.08%

Note: “Plant”, “Root”, “Stem”, “Leaf”, and “Boll” represent the fresh weight of the whole plant and each tissue, respectively. Values followed by different lowercase letters (a, b) indicate significant differences at the 5% probability level. MPH, mid-parent heterosis rate, MPH = (Hybrid trait value − Mean of parental trait values)/Mean of parental trait values × 100%; SPH, super-parent heterosis rate, SPH = (Hybrid trait value − Higher parent trait value)/Higher parent trait value × 100%. A value greater than 0 indicates positive heterosis, while a value less than 0 indicates negative heterosis.

**Table 3:** Dry weight of various tissues at the boll stage.

Plant Lines	Dry Weight (g)				
	Plants	Roots	Stems	Leaves	Bolls
GGK2	100.83 ± 8.22 a	4.39 ± 0.59 ab	20.64 ± 1.88 a	14.15 ± 0.87 ab	61.65 ± 6.54 a
XH	90.32 ± 6.88 a	4.00 ± 0.45 b	19.31 ± 1.92 a	11.08 ± 1.42 b	55.93 ± 3.58 a
GGK2 × XH	95.53 ± 5.54 a	5.99 ± 0.39 a	19.96 ± 1.07 a	17.13 ± 1.23 a	52.44 ± 3.57 a
MPH	−0.05%	42.87%	−0.07%	35.83%	−10.80%
SPH	−5.25%	36.42%	−3.28%	21.09%	−14.93%
GGK2	100.83 ± 8.22 b	4.39 ± 0.59 a	20.64 ± 1.88 ab	14.15 ± 0.87 b	61.65 ± 6.54 a
Y4	93.30 ± 7.15 b	4.62 ± 0.72 a	15.67 ± 1.10 b	15.85 ± 0.71 b	57.15 ± 7.22 a
GGK2 × Y4	149.51 ± 19.65 a	7.14 ± 1.33 a	27.07 ± 4.81 a	29.18 ± 4.74 a	86.12 ± 10.01 a
MPH	54.03%	58.45%	49.12%	94.51%	44.98%
SPH	48.28%	54.51%	31.17%	84.04%	39.69%
GGK2	100.83 ± 8.22 a	4.39 ± 0.59 a	20.64 ± 1.88 a	14.15 ± 0.87 b	61.65 ± 6.54 a
Y5	92.58 ± 4.27 a	4.82 ± 0.25 a	17.28 ± 1.03 a	14.04 ± 1.27 b	56.45 ± 2.64 a
GGK2 × Y5	115.40 ± 8.19 a	5.92 ± 0.75 a	22.57 ± 2.02 a	18.56 ± 0.87 a	68.36 ± 5.72 a
MPH	19.34%	31.72%	15.76%	28.48%	19.03%
SPH	14.46%	22.84%	9.33%	31.22%	10.88%

Note: “Plant”, “Root”, “Stem”, “Leaf”, and “Boll” represent the dry weight of the whole plant and each tissue, respectively. Values followed by different lowercase letters (a, b) indicate significant differences at the 5% probability level. MPH, mid-parent heterosis rate, MPH = (Hybrid trait value − Mean of parental trait values)/Mean of parental trait values × 100%; SPH, super-parent heterosis rate, SPH = (Hybrid trait value − Higher parent trait value)/Higher parent trait value × 100%. A value greater than 0 indicates positive heterosis, while a value less than 0 indicates negative heterosis.

**Table 4:** Agronomic traits of different cotton lines at the boll stage.

Plant Lines	Plant Height (cm)	Height of the First Fruiting Branch (cm)	Number of Fruiting Branches (a)	Number of Cotton Bolls (a)	Stem Diameter (mm)
GGK2	91.57 ± 0.57 b	34.15 ± 0.35 c	8.80 ± 0.18 b	11.13 ± 0.27 ab	10.02 ± 0.15 b
XH	101.46 ± 0.65 a	43.83 ± 0.28 a	8.60 ± 0.32 b	10.27 ± 0.61 b	10.63 ± 0.19 a
GGK2 × XH	98.89 ± 1.22 a	41.35 ± 0.43 b	9.93 ± 0.28 a	12.20 ± 0.4 a	9.66 ± 0.13 b
MPH	2.46%	6.06%	14.18%	14.02%	−6.39%
SPH	−2.54%	−5.66%	12.88%	9.58%	−9.09%
GGK2	91.57 ± 0.59 a	34.15 ± 0.35 a	8.80 ± 0.18 b	11.13 ± 0.27 a	10.02 ± 0.15 b
Y4	91.23 ± 0.93 a	32.85 ± 0.51 a	9.60 ± 0.19 a	11.47 ± 0.44 a	10.69 ± 0.18 a
GGK2 × Y4	82.62 ± 0.96 b	28.69 ± 0.51 b	8.27 ± 0.21 b	10.40 ± 0.34 a	10.12 ± 0.23 ab
MPH	−9.61%	−14.37%	−10.14%	−7.96%	−2.21%
SPH	−9.77%	−15.99%	−13.89%	−9.30%	−5.27%
GGK2	91.57 ± 0.59 a	34.15 ± 0.35 a	8.80 ± 0.18 b	11.13 ± 0.27 ab	10.02 ± 0.15 b
Y5	84.17 ± 0.56 b	30.50 ± 0.43 b	8.80 ± 0.20 b	10.07 ± 0.49 b	10.52 ± 0.16 b
GGK2 × Y5	93.13 ± 0.96 a	33.99 ± 0.29 a	10.00 ± 0.28 a	11.80 ± 0.42 a	11.24 ± 0.27 a
MPH	5.99%	5.17%	13.64%	11.32%	9.43%
SPH	1.71%	−0.45%	13.64%	5.99%	6.81%

Note: This table presents the agronomic traits of various cotton lines at the boll stage, including plant height, height to the first fruiting branch, number of fruiting branches, number of bolls, and stem diameter. The letters a, b, and c indicate significant differences at the 5% probability level. MPH, mid-parent heterosis rate, MPH = (hybrid trait value − average parental trait value)/average parental trait value × 100%; SPH, super-parent heterosis rate, SPH = (hybrid trait value − high-parent trait value)/high-parent trait value × 100%. Values greater than 0 indicate positive heterosis, while values less than 0 indicate negative heterosis.

**Table 5:** Yield traits of various cotton lines at maturity stage.

Plant Lines	No. Bolls (Plant^−1^)	Weight (Plant^−1^)	Weight (Boll^−1^)	Yield (kg·ha^−1^)	Lint Score (%)	Seed Index (g)
GGK2	8.15 ± 0.24 b	43.89 ± 1.87 a	5.36 ± 0.11 a	52.27 ± 2.23 a	0.39 ± 0.01 a	10.25 ± 0.07 b
XH	7.95 ± 0.30 b	42.32 ± 1.66 a	5.35 ± 0.14 a	50.40 ± 1.97 a	0.39 ± 0.01 a	11.15 ± 0.08 a
GGK2 × XH	9.9 ± 0.60 a	45.59 ± 2.81 a	4.62 ± 0.11 b	54.30 ± 3.35 a	0.37 ± 0.01 b	10.88 ± 0.15 a
MPH	22.98%	5.77%	−13.67%	5.77%	−4.94%	1.65%
SPH	21.47%	3.87%	−13.74%	3.87%	−4.85%	−2.42%
GGK2	8.15 ± 0.24 b	43.89 ± 1.87 a	5.36 ± 0.11 a	52.27 ± 2.23 a	0.39 ± 0.01 a	10.25 ± 0.07 c
Y4	9.10 ± 0.50 ab	46.83 ± 2.07 a	5.21 ± 0.10 ab	55.77 ± 2.46 a	0.34 ± 0.01 b	13.59 ± 0.07 a
GGK2 × Y4	10.00 ± 0.60 a	48.52 ± 3.24 a	4.85 ± 0.15 b	57.79 ± 3.86 a	0.38 ± 0.01 a	11.22 ± 0.08 c
MPH	15.94%	6.98%	−8.28%	6.98%	4.29%	−5.92%
SPH	9.89%	3.62%	−9.60%	10.56%	−3.17%	−17.44%
GGK2	8.15 ± 0.24 a	43.89 ± 1.87 a	5.36 ± 0.11 a	52.27 ± 2.23 a	0.39 ± 0.01 a	10.25 ± 0.07 c
Y5	8.55 ± 0.37 a	44.75 ± 1.64 a	5.29 ± 0.12 a	53.30 ± 1.96 a	0.34 ± 0.01 c	12.76 ± 0.11 b
GGK2 × Y5	8.45 ± 0.34 a	43.41 ± 1.55 a	5.16 ± 0.08 a	51.71 ± 1.85 a	0.36 ± 0.01 b	11.09 ± 0.05 a
MPH	1.20%	−2.04%	−3.03%	−2.05%	−2.83%	−3.61%
SPH	−1.17%	−2.99%	−3.73%	−1.08%	−9.64%	−13.09%

Note: This table presents the yield traits at maturity for each cotton line, including boll number per plant, yield per plant, boll weight per plant, yield per unit area, lint score, and seed index. Letters a, b, and c indicate significant differences at the 5% probability level. MPH, mid-parent heterosis rate, MPH = (mean trait value of the hybrid − mean trait value of the parents)/mean trait value of the parents × 100%; SPH, super-parent heterosis rate, SPH = (trait value of the hybrid − trait value of the better parent)/trait value of the better parent × 100%. Values greater than 0 indicate positive heterosis, while values less than 0 indicate negative heterosis.

**Table 6:** Fiber Quality of Various Cotton Germplasms.

Plant Lines	Length (mm)	Uniformity (%)	Strength (cN/Tex)	Elongation (%)	Micronaire
GGK2	30.02 ± 0.22 b	87.74 ± 0.23 a	27.94 ± 0.14 b	10.36 ± 0.22 b	4.52 ± 0.14 ab
XH	33.52 ± 0.27 a	88.32 ± 0.23 a	30.00 ± 0.21 a	8.98 ± 0.13 a	4.74 ± 0.15 a
GGK2 × XH	29.96 ± 0.16 b	86.76 ± 0.35 b	30.00 ± 0.40 a	11.46 ± 0.31 c	4.26 ± 0.07 b
MPH	−5.70%	−1.44%	3.56%	18.51%	−8.00%
SPH	−10.44%	−1.77%	0.00%	15.37%	−4.64%
GGK2	30.02 ± 0.22 b	87.74 ± 0.23 a	27.94 ± 0.14 a	10.36 ± 0.22 a	4.52 ± 0.14 a
Y4	32.44 ± 0.36 a	87.96 ± 0.44 a	27.98 ± 0.34 a	10.44 ± 0.30 a	4.60 ± 0.20 a
GGK2 × Y4	30.36 ± 0.32 b	86.76 ± 0.48 a	26.68 ± 0.42 b	10.86 ± 0.21 a	4.94 ± 0.12 a
MPH	−2.78%	−1.24%	−4.58%	4.42%	8.33%
SPH	−6.41%	−1.36%	−4.65%	4.02%	7.39%
GGK2	30.02 ± 0.22 b	87.74 ± 0.23 b	27.94 ± 0.14 b	10.36 ± 0.22 b	4.52 ± 0.14 a
Y5	31.28 ± 0.23 a	89.48 ± 0.37 a	29.48 ± 0.31 a	11.18 ± 0.20 a	4.46 ± 0.12 a
GGK2 × Y5	31.78 ± 0.21 a	88.28 ± 0.37 b	28.00 ± 0.21 b	10.92 ± 0.18 ab	4.82 ± 0.14 a
MPH	3.69%	0.37%	−2.47%	1.39%	7.35%
SPH	1.60%	−1.34%	−5.02%	−2.33%	6.64%

Note: This table presents the fiber quality of each cotton variety, including fiber length, uniformity, breaking tenacity, elongation at break, and micronaire value. The letters a, b, and c indicate significant differences at the 5% probability level. MPH, mid-parent heterosis rate, MPH = (F_1_ trait value − mean of parental traits)/mean of parental traits × 100%; SPH, super-parent heterosis rate, SPH = (F_1_ trait value − higher parent trait value)/higher parent trait value × 100%. Values greater than 0 indicate positive heterosis, while values less than 0 indicate negative heterosis.

## Data Availability

The original contributions presented in this study are included in the article. Further inquiries can be directed to the corresponding authors.

## Appendix A

**Table A1:** Determination of Pigment Content at the Boll Stage in Three Hybrid Combinations. Chl a. Determination of Chlorophyll a Content, Chl b. Determination of Chlorophyll b Content, Car. Determination of Carotenoid Content, Chl T. Determination of Total Chlorophyll Content, Chl a/b. Chlorophyll a/Chlorophyll b Ratio. Values followed by different lowercase letters (a, b) indicate significant differences at the 5% probability level.

Plant Lines	Chl a	Chl b	Car	Chl T	Chl a/b
GGK2	1.05 ± 0.11 a	0.32 ± 0.04 a	0.05 ± 0.01 a	1.36 ± 0.15 a	3.26 ± 0.05 b
XH	1.04 ± 0.10 a	0.32 ± 0.04 a	0.05 ± 0.01 a	1.35 ± 0.14 a	3.14 ± 0.12 b
GGK2 × XH	0.99 ± 0.07 a	0.30 ± 0.03 a	0.05 ± 0.01 a	1.30 ± 0.10 a	3.56 ± 0.15 a
MPH	−4.76%	−4.62%	−5.21%	−4.72%	11.32%
SPH	−5.07%	−5.30%	−7.84%	−5.12%	9.36%
GGK2	1.05 ± 0.11 a	0.32 ± 0.04 a	0.05 ± 0.01 a	1.36 ± 0.15 a	3.26 ± 0.05 a
Y4	1.09 ± 0.12 a	0.35 ± 0.06 a	0.04 ± 0.01 a	1.44 ± 0.18 a	3.31 ± 0.11 a
GGK2 × Y4	0.99 ± 0.01 a	0.28 ± 0.01 a	0.05 ± 0.01 a	1.26 ± 0.013 a	3.29 ± 0.12 a
MPH	−7.82%	−17.74%	8.32%	−10.19%	0.15%
SPH	−9.80%	−21.35%	3.04%	−12.61%	−0.61%
GGK2	1.05 ± 0.11 ab	0.32 ± 0.05 a	0.049 ± 0.01 a	1.36 ± 0.15 a	3.26 ± 0.05 b
Y5	0.87 ± 0.05 b	0.26 ± 0.020 a	0.06 ± 0.01 a	1.13 ± 0.07 a	3.41 ± 0.10 ab
GGK2 × Y5	1.23 ± 0.09 a	0.33 ± 0.03 a	0.06 ± 0.01 a	1.56 ± 0.12 a	3.75 ± 0.12 a
MPH	28.08%	14.09%	22.39%	24.84%	12.51%
SPH	17.34%	2.49%	15.18%	13.86%	9.86%
